# PM_2.5_ exposure as a risk factor for multiple sclerosis. An ecological study with a Bayesian mapping approach

**DOI:** 10.1007/s11356-020-10595-5

**Published:** 2020-09-07

**Authors:** Roberto Bergamaschi, Maria Cristina Monti, Leonardo Trivelli, Giulia Mallucci, Leonardo Gerosa, Enrico Pisoni, Cristina Montomoli

**Affiliations:** 1 Multiple Sclerosis Centre, IRCCS Mondino Foundation, via Mondino 2, 27100 Pavia, Italy; 2 Department of Public Health, Experimental and Forensic Medicine, University of Pavia, Pavia, Italy; 3 Department of Brain and Behavioural Sciences, University of Pavia, Pavia, Italy; 4 European Commission, Joint Research Centre (JRC), Ispra, Italy

**Keywords:** Multiple sclerosis, Epidemiology, PM_2.5_, Air pollution, Bayesian mapping, Ecological study

## Abstract

Some environmental factors are associated with an increased risk of multiple sclerosis (MS). Air pollution could be a main one. This study was conducted to investigate the association of particulate matter 2.5 (PM_2.5_) concentrations with MS prevalence in the province of Pavia, Italy. The overall MS prevalence in the province of Pavia is 169.4 per 100,000 inhabitants. Spatial ground-level PM_2.5_ gridded data were analysed, by municipality, for the period 2010–2016. Municipalities were grouped by tertiles according to PM_2.5_ concentration. Ecological regression and Bayesian statistics were used to analyse the association between PM_2.5_ concentrations, degree of urbanization, deprivation index and MS risk. MS risk was higher among persons living in areas with an average winter PM_2.5_ concentration above the European annual limit value (25 μg/m^3^). The Bayesian map revealed sizeable MS high-risk clusters. The study found a relationship between low MS risk and lower PM_2.5_ levels, strengthening the suggestion that air pollution may be one of the environmental risk factors for MS.

## Introduction

Multiple sclerosis (MS) is a complex disorder, likely arising from interactions between numerous genetic factors and several still incompletely clarified environmental factors (Thompson et al. 2018).

These environmental factors include diet and vitamin D levels (Ascherio and Munger 2007), both of which have been widely studied. However, air pollution, too, might influence the pathogenesis of immune and inflammatory processes in MS through various mechanisms, such as the development of oxidative stress and proinflammatory milieu (Veldhoen et al. 2008) (Odoardi et al. 2012), blood-brain barrier breakdown (Minagar and Alexander 2003; Kooij et al. 2010; Oppenheim et al. 2013), microglia activation (Calderón-Garcidueñas et al. 2002; Li et al. 2005) and vitamin D hypovitaminosis (Hosseinpanah et al. 2010).

Air quality is a major concern in northern Italy, especially in the Po Valley (*Pianura Padana*), a flat area particularly prone to high levels of particulate matter (PM). There are several reasons for this: high anthropogenic emissions, the natural geography of the area (with the Alps forming a barrier to the north) and its typically low wind speed that favours the formation and accumulation of pollutants.

The existence of possible links between air pollution and the epidemiology of MS has been investigated in few studies. It was found indeed an association between PM_10_ levels and MS prevalence (Gregory et al. 2008), as well as links with the occurrence of MS clinical relapses (Koski et al. 2003; Roux et al. 2017; Jeanjean et al. 2018) and MRI ‘activity’ (Bergamaschi et al. 2018).

A multicentre study in Italy found a 42% increase in hospital admissions for MS in Lombardy after elevated daily PM_10_ levels (Angelici et al. 2016). In another Italian study, the prevalence of MS in the north-eastern province of Padua (Veneto region) was demonstrated to be higher in urban areas with increased PM_2.5_ levels than in rural villages/localities (Tateo et al. 2019).

From an epidemiological point of view, it is appealing to analyse possible relationships between air pollution and MS occurrence in relatively small areas, with a view to enabling future research to focus on specific clusters of interest.

The present ecological study was conducted to investigate the spatial distribution of MS risk across the administrative areas of the province of Pavia (northern Italy), based on air pollution distribution (PM_2.5_ concentrations).

## Materials and methods

### Study population

MS cases were ascertained employing the sources and inclusion criteria already used in the previous research conducted by our group (Bergamaschi et al. 2006).

Briefly, the primary source was the Pavia Multiple Sclerosis Registry (PREMS) kept by the MS Centre at the C. Mondino Foundation in Pavia; this registry has been active since 1990 and has been part of the ‘Italian Multiple Sclerosis Registry’ since 2015. Data were also drawn from the data warehouse of the local Health Protection Agency (HPA), Department of Hygiene and Health Prevention of Pavia, specifically searching for exemption code (046.340) and the ICD-9-CM code for MS (340). Data were subsequently cleaned of duplicates.

The clinical documentation of the patients found through the HPA data (i.e. patients not followed by the MS Centre at the Mondino Foundation and not included in the PREMS) were reviewed by a senior neurologist (RB) to verify the validity of the diagnosis. Each patient’s life status was checked by consulting the HPA mortality registry.

All residents of the province of Pavia with a diagnosis of MS according to the 2010 revised McDonald diagnostic criteria (Polman et al. 2011) were included in the study. The mean delay in reporting MS cases was taken into account (Esbjerg et al. 1999) by setting 31 December 2016 as the prevalence day. On this day, the total count of MS patients in the province of Pavia was 927, and the overall prevalence of MS was 169.4 per 100,000 inhabitants (95% CI: 158.8–180.6) (Bergamaschi et al. 2019).

### Study area

The province of Pavia is one of the 12 provinces in the north-western Italian region of Lombardy. It covers an area of 2965 km^2^. The province is subdivided into 188 administrative areas called municipalities, and data from the 2017 inter-census ISTAT survey (http://demo.istat.it/pop2017/) reported 547,251 inhabitants (266,487 males and 280,764 females).

In the EU, local administrative units (LAUs) can be classified, for statistical stratification analysis purposes, according to their degree of urbanization (Eurostat 2018) as: 1-Cities (densely populated areas), 2-Towns and suburbs (intermediate density areas) and 3-Rural areas (thinly populated areas). Since Pavia and Vigevano are the only municipalities in the province of Pavia classifiable as cities (with 72,612 and 63,153 inhabitants, respectively), we re-classified the province’s LAUs into two categories: cities/towns and suburbs (LAU categories 1 and 2) vs rural areas (LAU category 3).

In addition, the deprivation index developed by Caranci et al. 2010 was used as a measure of socioeconomic status, calculated for municipalities and standardized at provincial level. This index is based on five main factors that represent the ‘multidimensionality of the social and material deprivation concept’ (namely, low education level, lack of employment, percentage of occupied rented houses, single-parent families, high housing density), and it provides accurate profiles of socioeconomic-health inequalities (Caranci et al. 2010).

### Environmental data source

Spatial information on pollutants was gathered from the database of the European Monitoring and Evaluation Programme (EMEP), a scientifically based and policy-driven programme under the Convention on Long-range Transboundary Air Pollution, signed by member states of the United Nations Economic Commission for Europe. The EMEP aims to cut down polluting emissions from several sources.

The database is constituted by gridded emissions and concentrations of several air pollutants (SOx, NH_3_, CO, NOx, PM_2.5_, PM_10_) in 0.1° × 0.1° (long-lat) resolution for the whole time series, from the year 2000 onwards. The EMEP grid domain covers the geographical area between 30°N-82°N latitude and 30°W-90°E longitude. The emission distribution is based on reported gridded emissions. Additional data were also obtained from the Emissions Database for Global Atmospheric Research https://edgar.jrc.ec.europa.eu.

In accordance with the purpose of the study, gridded data on PM_2.5_ for the province of Pavia were extracted. The grid was further subdivided into the province’s 188 municipalities. For each municipality, average seasonal (winter/summer) concentrations of PM_2.5_ were extracted and grouped by tertiles: lowest tertile 6.74–25 μg/m^3^, middle tertile 25.1–31.9 μg/m^3^ and highest tertile 32–46.6 μg/m^3^. The EU currently stipulates an annual PM_2.5_ limit value of 25 μg/m^3^ (https://www.eea.europa.eu/themes/air/air-quality-concentrations/air-quality-standards).

### Statistical analysis

Expected counts were calculated using direct standardization methods based on age- and sex-specific prevalence rates observed in the study area/period. Standardized morbidity ratios (SMRs) were calculated as ratios between observed and expected counts. To compare maximum likelihood estimates of prevalence rates and SMR differences, we used beta-binomial and gamma-Poisson distributions, respectively, in a hierarchical Bayesian fashion.

Cases were assigned the ecological characteristics (PM_2.5_ concentrations, degree of urbanization, deprivation index) of the geographical unit (municipality) they were resident in.

The SMR is unable to provide reliable spatial distribution of the disease on a small geographical grid, as high estimates are often obtained for small, sparsely inhabited areas. Hence, a Bayesian two-stage modelling approach was employed in order to draw MS relative risk (RR) maps. In the first stage, a Poisson log-linear model for count data was used (Agresti 2012); in the second stage, a log-linear hierarchical generalized mixed model (HGLMM) with reparametrized Besag York and Mollié random effects (BYM2) was implemented. The BYM2 method uses informative prior distributions (conditioning for the previous results) and both spatially structured and exchangeable error terms, thereby accounting for the degree of variability not explained by predictors in the first stage model (Besag et al. 1991; Riebler et al. 2016). The proposed final full Bayesian HGLMM model related the observed count of MS cases for each area to spatially structured random effects and PM_2.5_ levels, degree of urbanization and deprivation index.

We used medians of marginal posterior distributions of RR as estimators of the ‘true risk’. Moreover, to assess the presence of disease risk clusters and to evaluate our uncertainty about our risk estimates, we used posterior probabilities (PPs), PP being defined as the relative frequency of samples with RR > 1 for each municipality. The PP range (0–1) was divided into five intervals (< 0.10, 0.10–0.25, 0.25–0.75, 0.75–0.90, > 0.90). A PP value higher than or equal to 0.90 strongly indicated an ‘area of high risk’, while a PP value smaller than or equal to 0.10 strongly indicated an ‘area of low risk’. Similarly, PP values falling within the fourth interval (0.75–0.90) and second interval (0.10–0.25) merely indicated, respectively, areas of high and low risk of MS. As regards PP values in the central interval (0.25–0.75), the degree of evidence was considered insufficient to allow judgement. In conclusion, the map of PPs can be used to evaluate how confident we should be when we analyse area-specific RRs.

Calculations were carried out using statistical software, specifically the Stata 15, R version 3.6.1, Stan 2.17.3 and R packages.

## Results

### Distribution of PM_2.5_ concentrations in the province of Pavia

The mean concentrations of PM_2.5_ across the 7-year time series (2010–2016) were calculated for each municipality of the province.

**Fig. 1:**
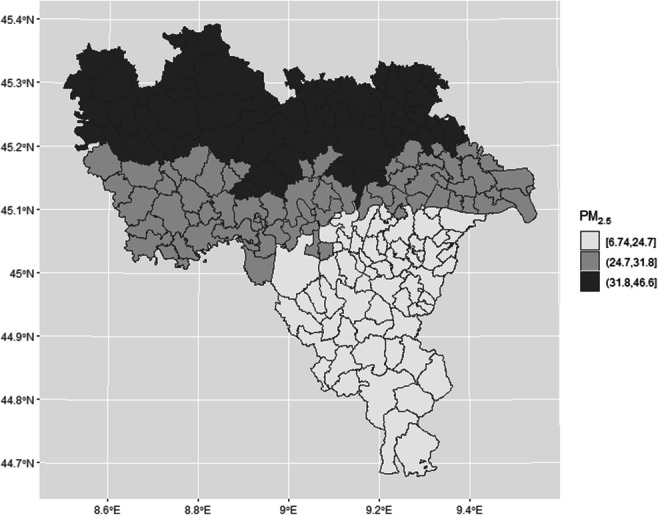
Average winter concentrations of PM_2.5_ across the 188 municipalities in the province of Pavia, divided into tertiles

The analysis focused on PM_2.5_ levels in winter, as it is the season with the highest pollutant concentrations. In terms of air pollution, the province of Pavia appeared to be divided into three strips (Fig. 1). The northernmost municipalities, being closer to the Milan area, demonstrated levels of PM_2.5_ within the range of the highest tertile. The southernmost municipalities in the rural area called *Oltrepò* appeared to be the least polluted, with levels of PM_2.5_ in the lowest tertile. Lying between these two strips, a central portion of the province showed intermediate PM_2.5_ levels, which were nevertheless above the European Commission threshold of 25 μg/m^3^*.* This portion of the territory includes both of the province’s two urban cities (*Pavia* and *Vigevano*).

### PM_2.5_ and MS risk

Geographical comparisons of specific MS prevalence estimates and SMRs between the area around Pavia (identified as a high MS risk cluster) and the low polluted area of *Oltrepò* (identified as a low MS risk cluster) revealed differences. Specifically, *Pavia and its hinterland* (the city and the 10 peri-urban bordering municipalities) had an MS prevalence of 196.4 per 100,000 inhabitants (95% CI: 171.8–224.6), while *Oltrepò* had an MS prevalence of 157.9 per 100,000 inhabitants (95% CI: 137.7–181.5) (*p* = 0.027). The SMR of *Pavia and its hinterland* was 1.14 (95% CI: 1.04–1.25), while in the *Oltrepò* area the SMR was 0.96 (95% CI: 0.87–1.03) (*p* = 0.0045).

A Bayesian model analysed area-specific RR of MS in relation to levels of PM_2.5_ exposure (divided into tertiles), degree of urbanization and deprivation index. It showed that MS risk, after adjustment for urbanization degree and deprivation index, was higher among persons residing in areas with a PM_2.5_ concentration in the middle tertile with respect to persons living in areas with a PM_2.5_ in the lowest tertile. The former had a 29% increased risk (RR = 1.29, 95% CI: 1.11–1.49, *p* value = 0.0001). Overall, MS risk was found to be higher in urban than in rural areas (RR = 1.16, 95%CI: 1.04–1.30, *p* value = 0.003). Deprivation index did not modify MS risk.

**Fig. 2:**
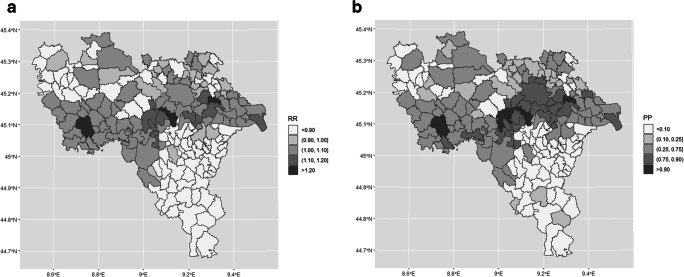
**a** Map of the relative risk (RR) of developing MS in association with average winter PM_2.5_ levels, after adjustment for degree of urbanization and deprivation index, across the 188 municipalities in the province of Pavia. Darker areas have higher RR values (gradients of grey). **b** Posterior probabilities (PPs) in association with average winter PM_2.5_ levels. The highest PPs were consistently identified in the areas also characterized by the highest RRs

The geographical distributions of RR and PP across the province of Pavia are depicted in Fig. 2. As seen in Fig. 2a, the municipalities associated with higher RRs (darker tones) are distributed around the centre of the map, in the area corresponding to intermediate winter concentrations of PM_2.5_. A similar PP distribution can be seen in Fig. 2b. This strip of territory roughly extends from the city of *Pavia* to the western portion of the province. The less polluted rural area of *Oltrepò* to the south showed consistently low RR levels.

## Discussion

The present study aimed to add to current knowledge on the multifactorial aetiology of MS, by exploring through an advanced statistical approach, the relationship between MS risk distribution and environmental air pollution in a well-defined geographical area.

To date, few studies have explored the distribution of MS in small geographical areas (Bergamaschi et al. 2006; Cocco et al. 2011). However, the province of Pavia, in Lombardy, has previously been investigated from this perspective. In 1976, it had an estimated MS prevalence of 16 cases per 100,000 inhabitants (Bergamaschi et al. 2006), and in 2000, this was found to have risen to 94 cases per 100,000 inhabitants (Bergamaschi et al. 2006). The latest estimate (169.4 per 100,000) (Bergamaschi et al. 2019) strongly indicates an increasing prevalence of the disease, in accordance with other literature data (Bezzini and Battaglia 2017; Grassivaro et al. 2019).

The increasing prevalence of MS can be explained in part by improved detection and longer survival of cases, which reflects the growing availability and efficacy of disease-modifying therapies. However, the increase in MS prevalence could also be explained by greater exposure to risk factors.

The availability of reliable estimates of MS distribution in small geographical areas would be crucial for identifying spatial clusters of MS risk. These clusters could then serve as the basis for ecological studies focusing on genetic and environmental factors. Unfortunately, the application of conventional statistical methods is probably unsuitable for analyses of this type. The main problem with a classical frequentist approach is that it produces ‘crude’ maps of the distribution of a disease that are strongly affected by random variation. This holds especially true in the presence of a relatively small number of cases that are in turn divided into numerous subgroups, e.g. small geographical units as in the present study. Under these conditions, the resulting frequentist map of the geographical disease distribution resembles a patchwork, being strongly subject to random errors. Therefore, these maps are difficult to interpret because the real effects we are interested in could be masked by random noise, and conversely, random noise could be mistaken for real effects. This issue can be overcome by building geographical distribution maps with a Bayesian method that separately models the random and the true variations. When the random noise from the final Bayesian map is filtered out, the map shows the true underlying variations in MS prevalence and can finally be taken as a reliable estimate of area-specific prevalence rates.

The clusters that emerged from Bayesian disease mapping in our study suggest that people living in the area with the lowest levels of pollutants (first tertile) have a low MS risk. The area in question, known as *Oltrepò,* is a hilly territory rich in vineyards, with no industrial settlements. With respect to rural dwellers, individuals residing in urban areas were found to have an increased risk of MS independently of PM_2.5_ levels. The highest risk was observed among individuals residing in more densely populated areas, especially those around the city of Pavia, which showed intermediate PM_2.5_ levels (the middle strip in Fig. 1).

We should ask why the highest MS risk was not observed in the more polluted northernmost municipalities. This finding could be partially due to the limitations of our study, which correction via the deprivation index failed to address completely. First, our analysis was based on formal residential addresses, which do not always correspond to where subjects actually live. Second, we did not collect data on occupation and therefore failed to take into account the fact that residents of a certain municipality may not necessarily spend most of their outdoor time there. Third, no data on indoor pollution were collected. Finally, although we analysed the prevalence and RR of MS, incidence data may be more useful in evaluating the relationship between disease risk and PM exposure.

## Conclusions

Albeit partly biased by the aforementioned issues, our study detected a lower risk of MS in individuals residing in rural areas with low levels of PM_2.5_, which suggests that reducing air pollution contributes to reducing the risk of developing MS.

In view of our epidemiological observation of an increasing trend in MS prevalence and our detection of clusters with an unexpected excess number of MS cases, we are currently carrying out specific analytical studies in these ‘high risk’ areas, with the aim of analysing multiple environmental factors, air pollution included, possibly related to the heterogeneous distribution of MS risk.

## Data Availability

The data that support the findings of this study are available on request from the corresponding author. The data are not publicly available due to privacy or ethical restrictions.
